# PINK1/Parkin-Dependent Mitochondrial Surveillance: From Pleiotropy to Parkinson's Disease

**DOI:** 10.3389/fnmol.2017.00120

**Published:** 2017-05-01

**Authors:** Francois Mouton-Liger, Maxime Jacoupy, Jean-Christophe Corvol, Olga Corti

**Affiliations:** ^1^Institut National de la Santé et de la Recherche Médicale, U1127Paris, France; ^2^Centre National de la Recherche Scientifique, UMR 7225Paris, France; ^3^Sorbonne Universités, UPMC Université Paris 06, UMR S 1127Paris, France; ^4^Institut du Cerveau et de la Moelle épinière, ICMParis, France; ^5^Department of Neurology, Institut National de la Santé et de la Recherche Médicale, Assistance-Publique Hôpitaux de Paris, CIC-1422, Hôpital Pitié-SalpêtrièreParis, France

**Keywords:** Parkinson disease, Pink1/Parkin, mitochondrial quality control, neuronal death, mitochondrial stress, neuroinflammation, UPRmt

## Abstract

Parkinson's disease (PD) is one of the most frequent neurodegenerative disease caused by the preferential, progressive degeneration of the dopaminergic (DA) neurons of the *substantia nigra* (SN) *pars compacta*. PD is characterized by a multifaceted pathological process involving protein misfolding, mitochondrial dysfunction, neuroinflammation and metabolism deregulation. The molecular mechanisms governing the complex interplay between the different facets of this process are still unknown. *PARK2*/Parkin and *PARK6*/PINK1, two genes responsible for familial forms of PD, act as a ubiquitous core signaling pathway, coupling mitochondrial stress to mitochondrial surveillance, by regulating mitochondrial dynamics, the removal of damaged mitochondrial components by mitochondria-derived vesicles, mitophagy, and mitochondrial biogenesis. Over the last decade, PINK1/Parkin-dependent mitochondrial quality control emerged as a pleiotropic regulatory pathway. Loss of its function impinges on a number of physiological processes suspected to contribute to PD pathogenesis. Its role in the regulation of innate immunity and inflammatory processes stands out, providing compelling support to the contribution of non-cell-autonomous immune mechanisms in PD. In this review, we illustrate the central role of this multifunctional pathway at the crossroads between mitochondrial stress, neuroinflammation and metabolism. We discuss how its dysfunction may contribute to PD pathogenesis and pinpoint major unresolved questions in the field.

## Introduction

Parkinson's disease (PD) is one of the most frequent neurodegenerative disorders, with more than six million people affected worldwide. Its cardinal motor symptoms (bradykinesia, muscle rigidity and tremor at rest) are caused by the preferential, progressive degeneration of the dopaminergic (DA) neurons of the SN *pars compacta*, and become manifest when more than 50% of these neurons are lost (Fearnley and Lees, 1991; Lang and Lozano, 1998; Damier et al., 1999). The presence of characteristic inclusion bodies enriched in the synaptic protein α-synuclein in neuronal processes and cell bodies, the Lewy neurites and Lewy bodies, are a second hallmark of the disease. Although often neglected, damage extends beyond the SN to other brain regions, including the dorsal IX/X motor nucleus, the raphe system, the locus coeruleus, the thalamus and amygdala, and in the most severe cases, neocortical areas (Lang and Lozano, 1998; Braak and Braak, 2000; Braak et al., 2004).

Why these neurons die in PD remains a mystery. One remarkable feature of the vulnerable neurons is their long, tortuous and frequently branching unmyelinated axon (Braak and Del Tredici, 2004). It was estimated that human DA neurons of the SN *pars compacta* have axons of an average length of 4.5 m, with an exceptionally complex arbor, forming between 1 and 2.4 million synapses in the striatum (Bolam and Pissadaki, 2012). Evidence from biology-based computational modeling suggests that such a large axonal architecture puts neurons under an extreme bioenergetic demand for propagation of action potentials, recovery of membrane potential and synaptic transmission (Pissadaki and Bolam, 2013). These unique features render these neurons highly susceptible to perturbations in energy supply. It is thus not surprising that mitochondrial dyshomeostasis emerged as one of the leading mechanisms suspected to play a role in PD pathogenesis.

## From toxins and mitochondrial complex I dysfunction to genes and mitophagy

The discovery of acute Parkinsonian syndromes caused by the DA neuron-specific mitochondrial neurotoxin MPTP (Exner et al., 2012), followed by reports of respiratory chain defects in brain and peripheral tissues from PD patients, first highlighted the role of mitochondria. More recent studies showed that, during aging, clonally expanded mitochondrial DNA (mtDNA) deletions accumulate at higher levels in human DA neurons of the SN compared to neurons in other brain regions (Bender et al., 2006; Kraytsberg et al., 2006). These changes appear to be compensated by an increase in the number of wild-type mtDNA copies in healthy individuals but not in PD patients (Dolle et al., 2016). However, the most compelling evidence for a central involvement of mitochondria in PD pathogenesis was provided by what we learned in the past decade about the functions of the proteins encoded by two genes mutated in autosomal recessive forms of PD: the RING/HECT hybrid E3 ubiquitin ligase Parkin and the mitochondrial serine/threonine kinase PINK1. Classical genetic complementation studies in *Drosophila melanogaster* suggested that the *PINK1* and *PARK2* (Parkin) genes are linked to a common pathway involved in mitochondrial maintenance and dynamics (Guo, 2012). Loss of their function in this organism causes profound morphological and functional alterations of the mitochondria, leading to flight muscle degeneration and cell death in specific DA neuron clusters. Work in mammalian cells extended these observations, demonstrating that PINK1 and Parkin jointly regulate the clearance of dysfunctional mitochondria by the autophagy-lysosomal pathway, a process termed mitophagy (Narendra et al., 2008, 2010; Geisler et al., 2010; Vives-Bauza and Przedborski, 2010; Pickrell and Youle, 2015).

**Figure 1:**
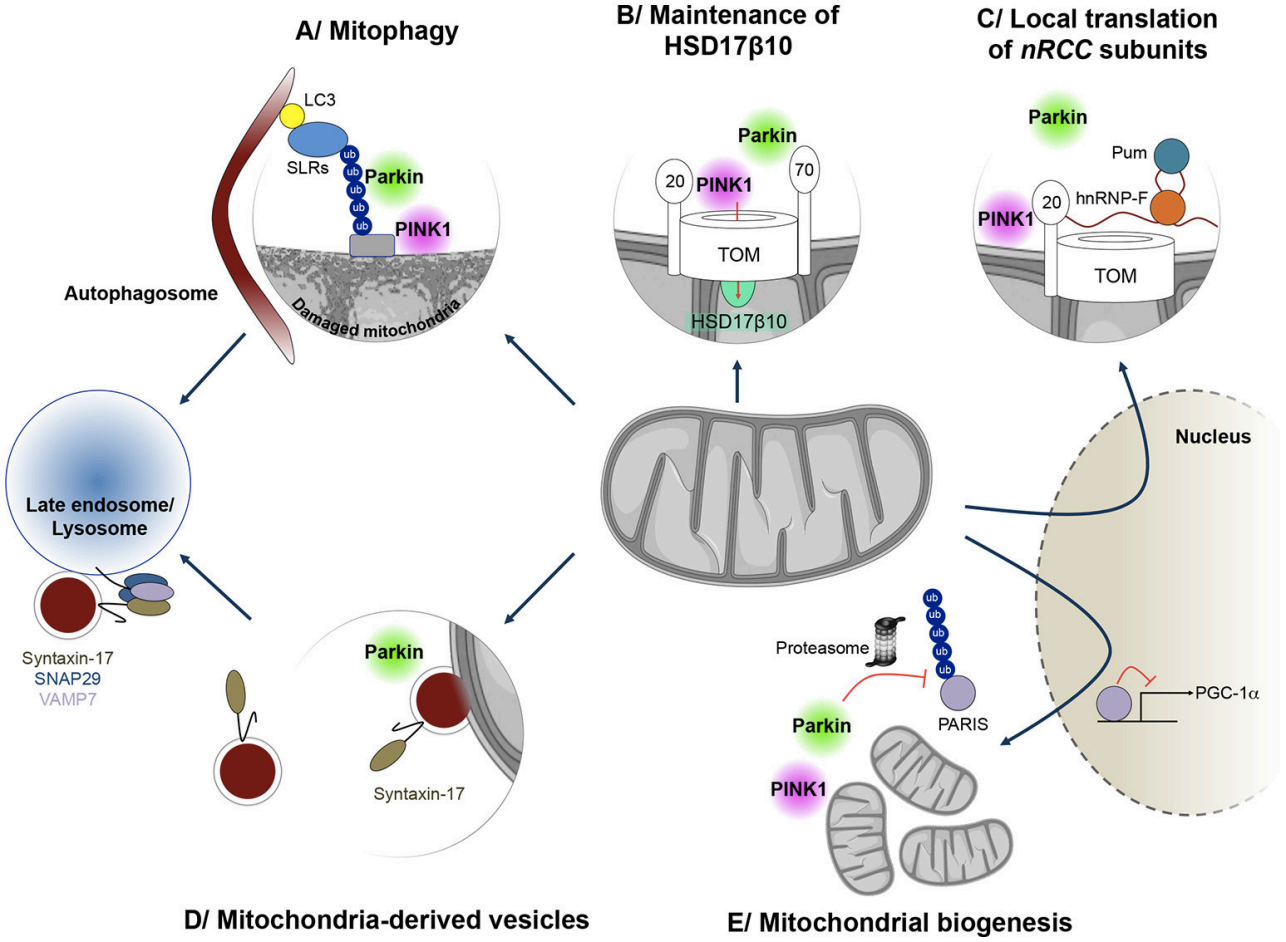
**Mitochondrial quality control mechanisms regulated by PINK1 and Parkin**. PINK1 and Parkin have cooperative roles in several processes implicated in mitochondrial surveillance. **(A)** When mitochondria are damaged, Parkin is recruited to the mitochondrial surface and activated by PINK1, to exert its E3 ubiquitin protein ligase function. Ubiquitin chains on mitochondria are preferentially bound by sequestosome 1/p62-like autophagy receptors (SLRs), such as Optineurin and NDP52, which in turn mediate autophagosome formation on the damaged mitochondrion, leading to mitophagy. **(B)** Parkin is also involved in maintenance of mitochondrial levels of the multifunctional PD-linked mitochondrial enzyme HSD17B10, possibly by promoting its import through the TOM complex **(C)** PINK1 and Parkin can also control localized translation of several mRNAs for nuclear-encoded subunits of respiratory chain complexes *(nRCC)*. Translationally repressed *nRCC* mRNAs are localized in a PINK1/Tom20-dependent manner to the mitochondrial outer membrane, where they are derepressed by Parkin through ubiquitin-dependent displacement of translation repressors, including Pumilio and Glorund/hnRNP-F. **(D)** PINK1 and Parkin also control the formation of so-called mitochondria-derived vesicles (MDVs), leading to the specific degradation of oxidized or damaged mitochondrial content by late endosomes/lysosomes. In this process, Syntaxin-17 is recruited to MDVs during budding, forming a ternary SNARE complex at the late endosome, together with SNAP29 and VAMP7, to mediate fusion. **(E)** Parkin also regulates the process of mitochondrial biogenesis by promoting the proteasomal degradation of the PGC-1α transcriptional repressor PARIS, in a PINK1-dependent manner.

Thanks to an impressive collective effort of the scientific community since its discovery in 2008, PINK1/Parkin-dependent mitophagy is now an extremely well characterized process (Figure 1A; Narendra et al., 2008; Pickrell and Youle, 2015; Yamano et al., 2016; Truban et al., 2017). It depends on the properties of the kinase PINK1, which acts as a molecular biosensor of mitochondrial protein import efficiency through the preprotein translocases of the outer (TOM) and inner (TIM) mitochondrial membranes (Jin et al., 2010; Greene et al., 2012; Bertolin et al., 2013). Mitochondrial depolarization impairs this process, leading to accumulation of PINK1 on the outer mitochondrial membrane, followed by recruitment and activation of Parkin through phosphorylation of a specific serine residue (Ser65) in both ubiquitin and the N-terminal ubiquitin-like domain of Parkin (Pickrell and Youle, 2015). This triggers a cascade of events, involving: (i) separation of the damaged organelle from the healthy network, by a mechanism dependent on the dynamin-related pro-fission GTPase, Drp1, and associated with cleavage of the inner membrane dynamin-like fusion GTPase, OPA1; (ii) ubiquitylation of a wide range of mitochondrial proteins in a Parkin-dependent manner; (iii) proteasomal degradation of some of these, including the profusion GTPases, Mfn1 and Mfn2; (iv) recruitment of autophagy receptor proteins and capture of the organelle by the autophagosome; and (v) mitochondrial degradation by the lysosome.

## Mitophagy dysfunction in Parkinson's disease: where do we stand?

There has been a lively debate about whether mitophagy is a quality control mechanism of relevance to cells and neurons, in particular under physiological conditions (Grenier et al., 2013). Two well-known examples illustrate the critical role of specific mitophagy programs in physiological conditions. The first one relates to the removal of mitochondria during the development of red blood cells, which underlies the function of these cells in oxygen transport (Sandoval et al., 2008). The second one concerns the elimination of paternal mitochondria in the fertilized oocyte, which provides the basis for maternal mtDNA inheritance in several species, including humans (Song et al., 2016; Zhou et al., 2016). In these specific programs, whole mitochondrial networks are destroyed. PINK1/Parkin-dependent mitophagy was discovered thanks to an artificial system based on the overproduction of a fluorescently tagged Parkin protein in a tumor-derived cell line devoid of Parkin, and the use of the protonophore CCCP to depolarize the whole mitochondrial network. In this artificial system, too, the whole mitochondrial network is destroyed and mitophagy can thus be recorded as proportion of cells without mitochondria. However, physiological mitophagy beyond specific developmental programs should rather be regarded as a mechanism that locally removes damaged fragments of the mitochondrial network, warranting the used of more sensitive techniques for its analysis. Not surprisingly, endogenous Parkin does not lead to clearance of the whole mitochondrial network in any cell type. Such an event is most unlikely to occur in neurons, which satisfy their energy requirements through oxidative phosphorylation (Bolanos, 2016) and are therefore less prone to lose their mitochondria than immortalized cells or tumor cells, mainly relying on glycolysis. Although recent studies support the possibility that neurons use glycolysis to sustain synaptic function under energy stress (Rangaraju et al., 2014; Jang et al., 2016; Ashrafi et al., 2017), or under pathological conditions associated with mitochondrial dysfunction (Requejo-Aguilar and Bolanos, 2016), it is likely that mitophagy pathways are more tightly controlled in neurons than in other cell types more inclined to switch to glycolytic metabolism. This may explain why some authors have been unable to observe mitophagy and questioned its existence in neurons (Sterky et al., 2011; Van Laar et al., 2011; Grenier et al., 2013; Rakovic et al., 2013).

Despite this controversy, Cai et al, showed that Parkin-dependent mitochondrial clearance occurs in the somatodendritic region of primary cortical neurons subjected to CCCP treatment, by using fluorescently-tagged Parkin and autophagosomal/lysosomal markers coupled with live imaging approaches (Cai et al., 2012). In a more physiological paradigm based on the local depolarization of the mitochondrial network in hippocampal neurons, using the mitochondrion-targeted photosensitizer mt-KillerRed, and the use of microfluidic devices for the investigation of neuronal subcompartments, mitophagic events were observed preferentially in the axons (Ashrafi et al., 2014). These events were associated with recruitment of exogenously expressed Parkin to dysfunctional mitochondria, and were abolished in PINK1-deficient neurons. Mitochondrion-targeted pH-sensitive fluorescent biosensors, such as mito-Keima, coupled to RNA interference approaches, have also proven particularly useful for detecting physiological mitophagy in cultured neurons, and linking it to endogenous PINK1 and Parkin (Bingol et al., 2014). Mito-Keima was recently used in DA neurons derived from induced pluripotent stem cells, revealing mitophagy impairment in neurons from patients with *PARK2* mutations (Suzuki et al., 2017). Such impairment may possibly underlie the mitochondrial defects reported in these cells by other authors (Chung et al., 2016).

These approaches hold promise for understanding the role of mitophagy in specific neuronal populations in living organism (Sun et al., 2015; McWilliams et al., 2016). Besides pioneering studies showing a specific decrease in mitochondrial protein turnover in *parkin* and *pink1* mutant flies (Vincow et al., 2013), there is little evidence that Parkin-dependent mitophagy is relevant and that its impairment causes neuronal death *in vivo*. In an attempt to address this issue, Pickrell and Youle recently investigated the effects of Parkin deficiency in a mouse model expressing a proofreading-deficient version of the mitochondrial DNA polymerase γ, associated with an age-dependent accumulation of mtDNA mutations (Mutator mice) (Pickrell and Youle, 2015). While, individually, Parkin-deficient mice and Mutator mice did not show a neurodegenerative phenotype, there was a nearly 40 % loss in DA neurons in the SN of aged Parkin-deficient Mutator mice compared to wild-type mice. Mitochondrial dysfunction was exacerbated by the loss of Parkin in this model, and was linked to a greater predicted pathogenicity score of mtDNA mutations. Surprisingly, the overall abundance and type (synonymous vs. nonsynonymous) of these mutations was unchanged. This study supports the idea that Parkin protects DA neurons against mitochondrial dysfunction, but it does not demonstrate that mitophagy is the underlying mechanism.

## Beyond mitophagy: PINK1 and Parkin regulate other mechanisms relevant to mitochondrial quality

Mitochondrial quality control refers to a series of coordinated mechanisms that evolved to preserve a population of healthy mitochondria in the cell, of which mitophagy represents an extreme facet (Rugarli and Langer, 2012; Shutt and McBride, 2013). These mechanisms are vital to neurons, as their alteration causes neurodegenerative diseases. It is becoming increasingly clear that PINK1 and Parkin regulate them more broadly.

The dynamics of the mitochondrial network is intimately linked to the maintenance of mitochondrial activities. It is associated with cycles of fusion and fission events, regulated by the fusion factors of the inner (OPA1) and outer mitochondrial membranes (Mfn1 and Mfn2), and by the fission GTPase Drp1. Mitochondrial membrane fusion allows redistribution of mitochondrial content and thus protection against accumulation of damaged components, such as mutant mitochondrial nucleoids. It constitutes a pro-survival compensatory response under stress conditions, ensuring optimal ATP production and preventing the autophagic removal of mitochondria (Tondera et al., 2009; Gomes et al., 2011; Shutt and McBride, 2013). Severe mitochondrial dysfunction inhibits mitochondrial fusion, resulting in mitochondrial fragmentation due to unopposed fission and subsequent mitophagy. There is substantial evidence that PINK1 and Parkin regulate mitochondrial dynamics by various direct and indirect mechanisms, including through the regulation of Mfn1/2 and Drp1 (Lutz et al., 2009; Gegg et al., 2010; Tanaka et al., 2010; Ziviani et al., 2010; Guo, 2012; Corti and Brice, 2013; Buhlman et al., 2014; Sun et al., 2015; Pryde et al., 2016).

Mitochondrial chaperones and proteases, such as hsp60, and AAA (ATPases associated with various cellular activities) proteases of the inner membrane and matrix, including i/m-AAA, Lon and ClpXP, operate from within the organelle to protect it against the accumulation of misfolded or damaged polypeptides (Rugarli and Langer, 2012; Voos et al., 2016). In addition, mitochondrial proteases regulate key proteolytic events at the crossroads of other mitochondrial quality control pathways: the processing of OPA1, which in addition to controlling fusion also regulates cristae structure, mitochondrial DNA maintenance and apoptosis (Ishihara et al., 2006; Anand et al., 2014; MacVicar and Langer, 2016); and the multistep cleavage of PINK1, which is central to its function in monitoring mitochondrial protein import efficiency (Jin et al., 2010; Meissner et al., 2011, 2015; Greene et al., 2012; Yamano and Youle, 2013; Thomas et al., 2014). Other mechanisms have evolved to promote the degradation of damaged mitochondrial components outside mitochondria, through the proteasomal and lysosomal pathways. The proteasome intervenes in cooperation with the AAA-ATPase p97/VCP/Cdc48 to degrade outer mitochondrial membrane proteins (Xu et al., 2011). This process plays a role during mitophagy, where it mediates the degradation of Mfn2 following ubiquitylation by Parkin, thereby preventing refusion of the damaged organelle with the functional network (Tanaka et al., 2010). More recently, an autophagy-independent vesicular pathway has been described, relying on the formation of small vesicles (MDVs) budding from the mitochondria and transporting specific protein cargo (Sugiura et al., 2014). MDVs were initially reported to shuttle the mitochondrial-anchored small ubiquitin-like modifier E3 ligase MAPL to peroxisomes (Neuspiel et al., 2008; Figure 1D). However, they were rapidly recognized to transport specific oxidized mitochondrial cargo to the endolysosomal compartment, including subunits of the pyruvate dehydrogenase complex and the COX1 component of mitochondrial complex IV of the respiratory chain (Soubannier et al., 2012a,b; McLelland et al., 2014). The formation of these vesicles was stimulated under conditions of oxidative stress and was shown to depend on PINK1 and Parkin (McLelland et al., 2014). The delivery of MDVs to the endosome/lysosome requires canonical soluble NSF attachment protein receptor (SNARE) machinery and a ternary complex between the SNARE proteins Syntaxin-17, SNAP29 (Soluble NSF Attachment Protein 29) and VAMP7 (Vesicular Associated Membrane Protein 7) (McLelland et al., 2016).

There is an intimate link between pathways of mitochondrial degradation and mitochondrial biogenesis (Ploumi et al., 2017). In addition to their role in degradative pathways, PINK1 and Parkin regulate different aspects related to mitochondrial biogenesis. The majority of the nearly 1500 mitochondrial proteins are encoded by the nuclear genome and imported into the organelle. Only 13 polypeptides of the respiratory chain are encoded by the mitochondrial DNA. Mitochondrial biogenesis is therefore a complex process, requiring appropriate coordination between transcription, translation and import of nuclear-encoded components, and expression of mitochondrial genes. Sophisticated regulatory mechanisms, associated with both the nucleus and mitochondria, evolved to ensure this process in response to growth signals or energy deprivation. Specific nuclear transcription factors, such as the nuclear respiratory factors, NRF1 and NRF2, and their partner Peroxisome proliferator-activated receptor γ coactivator 1α (PGC1α), control the expression of genes coding for mitochondrial proteins. Together, these master transcriptional regulators activate networks of genes encoding respiratory chain complex subunits, components of the mitochondrial import machineries, proteins involved in detoxification processes and factors regulating mitochondrial DNA maintenance. Parkin activates PGC-1α-dependent transcription by promoting the ubiquitin-dependent degradation of the Parkin Interacting Substrate (PARIS/ZNF746), a KRAB and zinc finger protein and transcriptional repressor of PGC-1α (Shin et al., 2011). PARIS/ZNF746 was found to be more abundant in the striatum and SN patients with autosomal recessive and sporadic PD, as well as in *PARK2* knock-out mice (Shin et al., 2011) Moreover, viral vector-mediated overexpression of PARIS/ZNF746 in the mouse SN led to DA neurodegeneration, which was prevented by concomitant Parkin or PGC-1α overexpression. Conversely, PARIS/ZNF746 gene silencing protected against mitochondrial biogenesis defects and DA neuron loss following *PARK2* inactivation in adult mice (Stevens et al., 2015). Recently, PINK1 was found to cooperate with Parkin in the regulation of PARIS/ZNF746 by phosphorylating it at serine residues 322 and 613, thereby priming it for ubiquitylation (Lee et al., 2017; Figure 1E).

The localized translation of mRNAs encoding mitochondrial proteins on the outer mitochondrial membrane has emerged as another mechanism of relevance to mitochondrial biogenesis, allowing coupling of translation to protein import into the organelle (Xu et al., 2011; Lesnik et al., 2015). Only a few of the molecular actors playing a role in this process have been identified. Among these, PINK1 and Parkin cooperate in the mitochondrial targeting and local derepression of mRNAs encoding specific respiratory chain subunits, by an only partially elucidated mechanism, involving anchoring of the mRNAs to the TOM complex, displacement of translational inhibitors and recruitment of translational activators (Gehrke et al., 2015; Figure 1C). We have recently proposed a role of Parkin in the biogenesis of a specific mitochondrial protein, the hydroxysteroid dehydrogenase HSD17B10 (Bertolin et al., 2015). HSD17B10 is a multifunctional matrix enzyme essential for mitochondrial integrity and cell survival (Rauschenberger et al., 2010; Yang et al., 2014). Its levels were found to be lowered in the ventral midbrain of PD patients, and in the brains of mice intoxicated with MPTP (Tieu et al., 2004) or deficient for Parkin (Bertolin et al., 2015). On the other hand, overproduction of HSD17B10 protected against DA neuron loss triggered by MPTP intoxication (Tieu et al., 2004), suggesting that depletion of HSD17B10 contributes to PD pathogenesis. We showed that Parkin maintains HSD17B10 levels in mitochondria, possibly by promoting its import through the TOM machinery by a ubiquitin-dependent mechanism (Bertolin et al., 2015; Figure 1B).

Some mitochondrion-protective mechanisms are controlled by PINK1 independently of Parkin. For example, work in Drosophila and mouse models highlighted a specific role of PINK1 in the regulation of the activity of the mitochondrial NADH:ubiquinone oxidoreductase complex of the electron transport chain (complex I). PINK1-deficiency in these models led to primary mitochondrial complex I defects (Morais et al., 2009), associated with loss of phosphorylation of the complex I subunit, NDUFA10, at Ser250 (Morais et al., 2014). According to Morais and colleagues (Morais et al., 2014), this phosphorylation was sufficient to rescue complex I activity and mitochondria-related phenotypes in *pink1* null mutant flies, as well as in cells from *PINK1* knockout mice and patients with *PINK1* mutations. In an independent study, similar effects were observed following transgenic overexpression of NDUFA10 or its co-chaperone *sicily* in *pink1* null mutant flies (Pogson et al., 2014). However, the requirement for NDUFA10 phosphorylation was not confirmed. In addition, overexpression of NDUFA10 or *sicily* did not restore phenotypes in *parkin* null mutant flies, suggesting that PINK1 acts independently of Parkin in maintenance of complex I activity. Accordingly, strategies bypassing or restoring complex I activity, i.e., through expression of the Ndi1p NADH oxidoreductase from *Saccharomyces cerevisiae* or overexpression of NDUFA10 (Vilain et al., 2012; Morais et al., 2014; Pogson et al., 2014), attenuate phenotypes in *pink1* but not *parkin* mutant flies. Similarly, Vitamin K2 restored mitochondrial transmembrane potential and ATP levels in *pink1* mutants and in flies with lower levels of mitochondrial complex I components, presumably by functioning as the mitochondrial electron carrier ubiquinone (Vos et al., 2012) Surprisingly, though, Vitamin K2 was also efficient toward *parkin* loss-of-function phenotypes in this study.

Finally, early studies linked PINK1 with phosphorylation of the mitochondrial chaperone, tumor necrosis factor (TNF) receptor-associated protein TRAP1, (Pridgeon et al., 2007) and the mitochondrial intermembrane space serine protease, HtrA2 (Plun-Favreau et al., 2007). Complementary studies in Drosophila confirmed genetic interaction between each of these proteins and PINK1, although not always with consistent results (Tain et al., 2009; Costa et al., 2013; Zhang et al., 2013). Of note, while ubiquitin and the ubiquitin-like domain of Parkin have become uncontested phosphorylation substrates of PINK1, based on the most recent discoveries supported by structural studies (Caulfield et al., 2015; Dove et al., 2015), the significance of other putative PINK1 substrates remains to be clarified.

## From simple to complex: toward an integrated analysis of the cellular response to mitochondrial stress and its regulation by PINK1 and Parkin

Remarkable studies in the simple eukaryotic model organisms *Caenorhabditis (C.) elegans* and yeast, have pinpointed the intimate connection between mitochondrial dysfunction and cytosol- and nucleus-dependent retrograde signaling responses aimed at recovering mitochondrial activity and protecting the cell and organism against premature death (Liao and Butow, 1993; Butow and Avadhani, 2004; Wang et al., 2008; Nargund et al., 2012; Haynes et al., 2013; Liu et al., 2014; Pellegrino et al., 2014; Palikaras et al., 2015; Wang and Chen, 2015; Wrobel et al., 2015). These responses are elicited by various stimuli that impinge on mitochondrial activity. These include the mitochondrial toxins paraquat and antimycin (Nargund et al., 2012; Liu et al., 2014; Pellegrino et al., 2014), pathogens, such as Pseudomonas aeruginosa (Liu et al., 2014; Pellegrino et al., 2014), and mutations in genes encoding key mitochondrial components, such as the mitochondrial m-AAA protease SPG-7 (Nargund et al., 2012; Pellegrino et al., 2014), the adenine nucleotide translocase ANT (Wang and Chen, 2015), or subunits of the mitochondrial import machineries (Nargund et al., 2012; Wrobel et al., 2015). These conditions mobilize a number of partially overlapping protective programs, extending from the coordinated activation of mitophagy and biogenesis to the mitochondrial unfolded protein response (UPR^mt^) and parallel cytosolic proteostatic pathways (Baker et al., 2012; Delaney et al., 2013; Liu et al., 2014; Wang and Chen, 2015; Wrobel et al., 2015). The UPR^mt^ is one of the best characterized of these programs. It was initially described in mammalian cell models depleted for the mitochondrial genome or accumulating misfolded proteins within the organelle (Zhao et al., 2002; Runkel et al., 2013; Arnould et al., 2015). Here, it was associated with the transcriptional upregulation of nuclear-encoded mitochondrial proteases and chaperones. In recent years, the UPR^mt^ received a lot attention in *C. elegans* models, and we learned that it has a much broader scope than the reestablishment of mitochondrial proteostasis. A network of more than 400 genes induced by mitochondrial dysfunction, linked to protein folding capacity and quality control, mitochondrial biogenesis, detoxification processes, metabolic reprogramming and innate immunity, has been uncovered in this organism (Nargund et al., 2012, 2015; Haynes et al., 2013; Wu et al., 2014). Defects in this response affect development and survival under conditions of mitochondrial stress (Haynes et al., 2010; Nargund et al., 2012). Moreover, several studies linked UPR^mt^ activation associated with mitochondrial dysfunction with organismal lifespan extension, although whether UPR^mt^ is indispensable for increased longevity is debated (Durieux et al., 2011; Houtkooper et al., 2013; Schieber and Chandel, 2014; Schulz and Haynes, 2015).

Work in *C. elegans* has also led to the identification of several regulatory components of the UPR^mt^ pathway, including the mitochondrial protease ClpP, the mitochondrial peptide transporter HAF-1, the ubiquitin-like protein UBL-5 and the homeobox transcription factor DVE-1 (Haynes et al., 2013; Schulz and Haynes, 2015). Three RNAi screens from independent laboratories identified the bZip transcription factor, Activating Transcription Factor Associated with Stress 1 (ATFS-1), as a key actor in UPR^mt^ activation (Haynes et al., 2010; Runkel et al., 2013; Liu et al., 2014). ATFS-1 carries both an N-terminal mitochondrial targeting signal and a nuclear localization signal. In unchallenged cells, it is constitutively imported into functional mitochondria and quickly degraded in the matrix by the Lon protease. However, under mitochondrial stress, mitochondrial protein import is attenuated and ATFS-1 accumulates in the cytosol and traffics to the nucleus, where it activates the expression of UPR^mt^ genes (Nargund et al., 2012, 2015). Thus, analogously to PINK1, ATFS-1 senses mitochondrial stress in terms of loss of mitochondrial import efficiency, and translates it into a multilevel protective response. Mitochondrial import has thus emerged as a central step used by the cell to monitor mitochondrial function and link it to the regulation of stress and metabolism, across distant species (Nargund et al., 2012; Rainbolt et al., 2013; Harbauer et al., 2014; Wang and Chen, 2015; Wrobel et al., 2015). However, little is known about the mechanisms underlying the orchestration and reciprocal articulation of the different branches of the response to mitochondrial dysfunction, particularly in mammalian cells. Remarkably, there is evidence that PINK1/Parkin-dependent mitophagy is activated by the accumulation of misfolded proteins in the mitochondrial matrix (Jin and Youle, 2013), the *princeps* condition linked to UPR^mt^ activation in mammalian cells (Zhao et al., 2002). Thus, there is crosstalk between mitophagy and the UPR^mt^, raising the question of the molecular mechanisms underlying the common activation of these pathways during mitochondrial stress. Considering the strategic position of PINK1 at the TOM complex, it may be more broadly involved in mitochondrial stress signaling, possibly by controlling the activity of as yet unknown regulatory components. Notably, Fiorese et al. (2016) recently identified the transcription factor ATF5 as a possible functional homologue of ATFS-1, mediating the UPR^mt^ in mammalian cells (Fiorese et al., 2016). This opens the way for investigation of the possible regulation of ATF5 by PINK1/Parkin-dependent mechanisms.

Finally, we have learned another major lesson from studies of the UPR^mt^ in *C. elegans*: mitochondrial stress is intimately connected with the induction of genes encoding innate immunity components, such as anti-microbial peptides and C-type lectins (Melo and Ruvkun, 2012; Nargund et al., 2012; Pellegrino et al., 2014). Conversely, pathogens that perturb mitochondrial function trigger a protective UPR^mt^ response. Together with strong emerging links between PINK1 and Parkin and the regulation of pathogen response pathways in *C. elegans* and mammals (Mira et al., 2004; Manzanillo et al., 2013; Chopra et al., 2014; Kirienko et al., 2015), these observations warrant a detailed analysis of the role of PINK1/Parkin-dependent mitochondrial quality control in cells of the immune system, particularly in the central nervous system, where their dysfunction may contribute to neurodegeneration.

## A regulatory hub at the intersection between mitochondria and neuroinflammation

Neuronal death in PD is accompanied by considerable neuroinflammation and intrinsically-driven glial cell changes (Halliday and Stevens, 2011). In addition to their role in inflammation, which is often interpreted as a secondary phenomenon, glial cells may actively contribute to the degeneration of the nigrostriatal pathway and hasten the progression of PD. During the last 20 years, a wealth of new knowledge has deepened our understanding of the role of this mechanism in PD. Brain neuroinflammation in PD is characterized by excessive activation of microglial cells, as revealed in brains post-mortem, and highlighted by positron emission tomography scans of the inflammatory marker, PK11195. Furthermore, cytokines accumulate in the cerebrospinal fluid and serum of PD patients, (McGeer et al., 1988; Gerhard et al., 2006; Hirsch and Hunot, 2009), as in most experimental murine models of PD (Mogi et al., 2000; Gao et al., 2002). Moreover, aggregated α-synuclein, released by neurons, activates microglial cells to produce pro-inflammatory mediators (Hirsch and Hunot, 2009). Furthermore, genome-wide association studies linked common variants in immune-related regions to PD risk, and several genes mutated in monogenic PD forms modulate inflammatory pathways. However, the intracellular signaling mechanisms underlying aberrant neuroinflammation in PD are still poorly understood.

Healthy mitochondria have been shown to orchestrate glial cell reactions, by providing a platform for signaling pathways involved in innate immunity. Mitochondrial ROS (mtROS), mitochondrial antiviral signaling protein (MAVS) and mitochondrial damage-associated molecular patterns (mtDNA, mtTFA, N-formyl peptides, cardiolipin) play key roles in the activation of innate immune response following infection or tissue damage (West et al., 2011). There is mounting evidence that mitochondrial quality control (from dynamics, to biogenesis and mitophagy) regulates innate immune responses, primarily by maintaining a functional cohort of mitochondria within the cell (Cherry and Piantadosi, 2015; Khan et al., 2015; Lazarou, 2015). In a remarkable recent paper, Matheoud et al. demonstrated that, in response to cellular stress, self-antigens can be extracted from mitochondria via the MDV pathway and presented at the cell surface to trigger immune responses. This mechanism, called mitochondrial antigen presentation (MitAP), is repressed by PINK1 and Parkin. This work supports a non-cell-autonomous model in which autoimmune mechanisms contribute to the destruction of DA neurons in PD (Matheoud et al., 2016). On the other hand, mitochondria are vulnerable to damage triggered by infectious processes, hinting at an intricate crosstalk between inflammation and PINK1/Parkin-depedent mitochondrial quality control programs.

Historically, *PARK2*/Parkin was firstly associated with increased susceptibility to infection by mycobacteria, such as M. leprae and M. tuberculosis (Mira et al., 2004; Manzanillo et al., 2013; Chopra et al., 2014). Parkin modulates the host response to these pathogens by promoting their clearance via ubiquitin-mediated autophagy (Manzanillo et al., 2013). Parkin and PINK1 expression is also stimulated by viruses, such as hepatitis B virus (HBV) and hepatitis C virus (HCV) (Kim et al., 2013a,b). HBV and HCV promote mitochondrial translocation of Parkin and Parkin-dependent mitophagy (Kim et al., 2013a,b; Hara et al., 2014; Khan et al., 2016). In HBV infection, Parkin interacts with the mitochondrial adaptor protein MAVS, promoting its modification by unanchored linear polyubiquitin chains and triggering an antiviral signaling cascade associated with interferon β production (Khan et al., 2016). Taken together, these studies suggest that PINK1 and Parkin modulate susceptibility to infection, warranting further work aimed at evaluating in how far this mechanism contributes to PD risk. Evidence linking another PD gene to the interferon response, *LRRK2* (leucine-rich repeat kinase-2), reinforces this idea (Gardet et al., 2010).

Several groups reported that Parkin regulates the NF-κB-dependent inflammatory pathway by different mechanisms (Henn et al., 2007; Sha et al., 2010). Henn et al. (2007) were the first to pinpoint such a link, suggesting that it may contribute to the neuroprotective properties of Parkin (Henn et al., 2007). Accordingly, *PARK2* knockout mice exhibited increased susceptibility to neuronal loss triggered by the bacterial endotoxin, lipopolysaccharide (LPS), know to participate in IKKβ-NF-κB activation (Frank-Cannon et al., 2008). Moreover, Parkin-deficiency was associated with excessive cytokine production, such as TNFα, interleukin (IL)-6 or monocyte chemoattractant protein-1 (MCP-1) production, following LPS treatment either *in vitro* or *in vivo* (Tran et al., 2011; de Leseleuc et al., 2013). Several molecular mechanisms may underlie these regulations, including ubiquitylation of key components of the NF-κB pathway, such as IKKγ and TNF receptor-associated factor 2 (TRAF2) (Henn et al., 2007) or 6 (TRAF6) (Chung et al., 2013). Emerging studies suggest that this Parkin-NF-κB interaction counterbalances the action the NLRP3-inflammasome, a proinflammatory multiprotein complex activated by a number of endogenous and exogenous stimuli and leading to the caspase 1-mediated maturation of the cytokines, IL-1β and IL-18 (Baroja-Mazo et al., 2014; Zhong et al., 2016). A number of compelling studies have linked NLRP3-inflammasome activation to neuroinflammation in several neurodegenerative disease (Heneka et al., 2013; Malhotra et al., 2015). There is also evidence that NLRP3 could be activated by fibrillar α-synuclein in innate immune cells, suggesting an involvement in PD pathogenesis (Codolo et al., 2013). In agreement with this hypothesis, a recent study highlighted dopamine as an endogenous inhibitor of NLRP3 inflammasome activation, by a mechanism involving dopamine receptor D1-receptor signaling and microglial cells (Yan et al., 2015). NF-κB not only primes the inflammasome for activation by inducing the expression of pro-IL-1β and NLRP3 (Guo et al., 2015), but it also prevents excessive NLRP3 activation by promoting Parkin-dependent mitophagy (Zhong et al., 2016).

Interestingly, inflammasome activation requires NLRP3 recruitment to the endoplasmic reticulum (ER)-mitochondria interface (Zhou et al., 2011), possibly through the MAVS adaptor protein (Subramanian et al., 2013), and ER-derived calcium may be central to NLRP3 activation (Lee et al., 2012; Rossol et al., 2012). We have recently shown that Parkin-deficiency or dysfunction in knockout mice and *PARK2* PD patients exacerbates the ER-mitochondria interface, leading to excessive calcium transfer between these organelles (Erpapazoglou and Corti, 2015; Gautier et al., 2016). This provides a mechanism by which Parkin might modulate NLRP3 activity.

Mitochondrial dynamics also regulates the generation of pro-inflammatory mediators in activated microglial cells. Mitochondrial fission is induced in LPS-stimulated microglial cells, possibly contributing to ROS production and induction of pro-inflammatory cytokines through the activation of NF-κB and MAPK signaling (Park et al., 2013). Moreover, impairment of mitochondrial fission in macrophages triggered by Drp1 gene silencing may modulate the assembly and activation of the NLRP3 inflammasome in response to nigericin (Park et al., 2015) or RNA virus exposure (Wang et al., 2014). Mitochondrial localization of Drp1, and its phosphorylation state on serine 637, are key steps in all these regulations. In addition, both Mfn1 and Mfn2 positively regulate antiviral responses, in particular those mediated by RLR-MAVS signaling (Yasukawa et al., 2009; Onoguchi et al., 2010). Ichinohe et al. showed that Mfn2 mitigates the association between MAVS protein and NLRP3, thereby enhancing inflammasome activation (Ichinohe et al., 2013). Various functional links have been reported between PINK1 and Parkin and components of the mitochondrial fusion/fission machinery (Gegg et al., 2010; Tanaka et al., 2010; Ziviani et al., 2010; Muller-Rischart et al., 2013; Buhlman et al., 2014). It is likely that these interactions will turn out to participate in the modulation of innate immune and inflammatory pathways.

Altogether, these studies strongly support a role for the PINK1/Parkin pathway in the regulation of innate immunity and inflammation. One key question that will have to be addressed in the future is whether deregulation of the PINK1/Parkin pathway in immune cells has a deleterious impact on DA neurons. The establishment of co-cultures systems, integrating pre-activated microglia from *PARK2* or *PINK1* knockout mouse models with primary embryonic DA neurons may represent a preliminary step toward a more global analysis of these interactions in PD.

## The good mitophagy, the bad diabetes and their link to PD

It is well established that DA neurons and glia are highly sensitive to metabolic modifications, such as abnormalities in lipid biology and changes linked to type 2 diabetes mellitus (T2DM). DA neurons of the SN have distinct phospholipid metabolism compared with other brain region neurons (Ross et al., 1998), and express insulin and leptin receptors (Figlewicz et al., 2003). Insulin receptors of DA neurons act as key regulators of DA neurotransmission by increasing DA transporter activity and enhancing clearance of synaptic DA (Davis et al., 2011; Speed et al., 2011). Impaired insulin signaling aggravates brain dysfunction related to altered DA homeostasis (Carvelli et al., 2002; Garcia et al., 2005). Moreover, detrimental inflammatory conditions, resulting from high glucose concentrations, have been reported to induce DA neurons death in animal models of PD (Cai, 2009; Morris et al., 2010; Machado et al., 2011). Conversely, immune activation rapidly and substantially enhances metabolic outputs in immune cells (Rodriguez-Prados et al., 2010). In microglial cells, these metabolic changes are generally accompanied by marked modifications in the functional organization and morphology of the mitochondrial network (Banati et al., 2004). In PD patients, the impact of metabolic disorders is still a matter of debate. Epidemiologic evidence links lower levels of low-density lipoprotein cholesterol and fatty acids (Huang et al., 2008) or T2DM (Cereda et al., 2013) with increased risk of PD, or with a modifying effect on PD-related phenotypes (Cereda et al., 2012). At the same time, patients with PD have been consistently reported to be underweight compared with healthy controls. This discrepancy is likely due to premorbid weight loss, which begins years prior to first symptoms (Chen et al., 2003).

*PARK2* knockout mice are refractory to high fat diet (HFD)-induced weight gain and insulin resistance. A similar pattern of response was found in blood cells from patients with *PARK2* mutations, which exhibit a limited ability to absorb fat (Kim et al., 2011). Similarly, loss of PINK1 in β-cell impairs glucose uptake, increases basal insulin secretion and improves glucose tolerance in mice (Deas et al., 2014). Though surprising, based on the overall protective properties of the PINK1/Parkin pathway, these effects may be explained by tissue-specific differences in expression: Parkin and PINK1 protein levels were found to be increased in blood vessels of HFD mice (Wu et al., 2015), whereas a significant loss of Parkin was found in the SN of these mice (Khang et al., 2015). In SN of HFD mice, Parkin deficiency led to accumulation of the Parkin substrate, PARIS, and a reduction of PGC-1α (Khang et al., 2015), which in addition to regulating mitochondrial biogenesis, is also known to act as a powerful regulator of systemic metabolic homeostasis (Lin et al., 2004). Overexpression of PGC-1α in cell models decreased lipid-droplet accumulation and increased mitochondrial fatty acid oxidation and downregulation of PINK1 abolished these effects (Choi et al., 2014). Several *in vitro* and *in vivo* PD models support a neuroprotective effects of PGC-1α and its partner PPARγ (the peroxisome proliferator-activated receptors γ), a major player in lipogenesis (Chaturvedi and Beal, 2008; Corona and Duchen, 2015; Zheng et al., 2017). Altogether, this literature suggests the PINK1 and Parkin play a coordinated role with PGC-1α in metabolism regulation. However, it remains to be determined in how far this function is linked to the role of these proteins in mitochondrial quality control, rather than to direct effects on lipid homeostasis (Kim et al., 2011).

Notably, a recent study suggested that the metabolic syndrome caused in mice by deletion of the T2DM susceptibility gene *TP53INP1* (tumor protein 53-induced nuclear protein 1), involved in tumor suppression and regulation of autophagy, is due to impaired PINK1/PARKIN-mediated mitophagy, associated with oxidative stress and chronic inflammation (Seillier et al., 2015). This work reinforces the link between dysfunction of PINK1/Parkin-dependent mitochondrial control, diabetes and PD.

## Conclusion

Since the discovery of the mitochondrial neurotoxin MPTP in the early 1980s, the idea that mitochondria play a central role in the physiopathology of PD has gained tremendous strength over the last 10 years, through the knowledge acquired on the functions of two proteins involved in autosomal recessive PD forms, PINK1 and Parkin. The body of literature discussed in this review illustrates the multifunctional nature of these proteins, highlighted by their cooperative regulation of a number of aspects related to mitochondrial quality control, of which mitophagy is the most emblematic. Despite vivid debates on the relevance of this process to neurons, several studies taking advantage of fluorescent reporters have demonstrated its occurrence in neuronal primary cultures. In addition to mitophagy, PINK1 and Parkin co-regulate mitochondrial dynamics, the MDV pathway and mitochondrial biogenesis, but the mechanisms by which they orchestrate this gradual response in mammalian cells are poorly understood. Future studies will have to explore the possibility that PINK1 and Parkin are more broadly involved in mitochondrial stress signaling, considering their pivotal position at the crossroads of different pathways protecting against mitochondrial dysfunction. This implies a better understanding of the interrelations between the different branches of the mitochondrial stress response and their possible regulation by PINK1/Parkin-dependent mechanisms in proximity of the mitochondrial import machinery.

**Figure 2:**
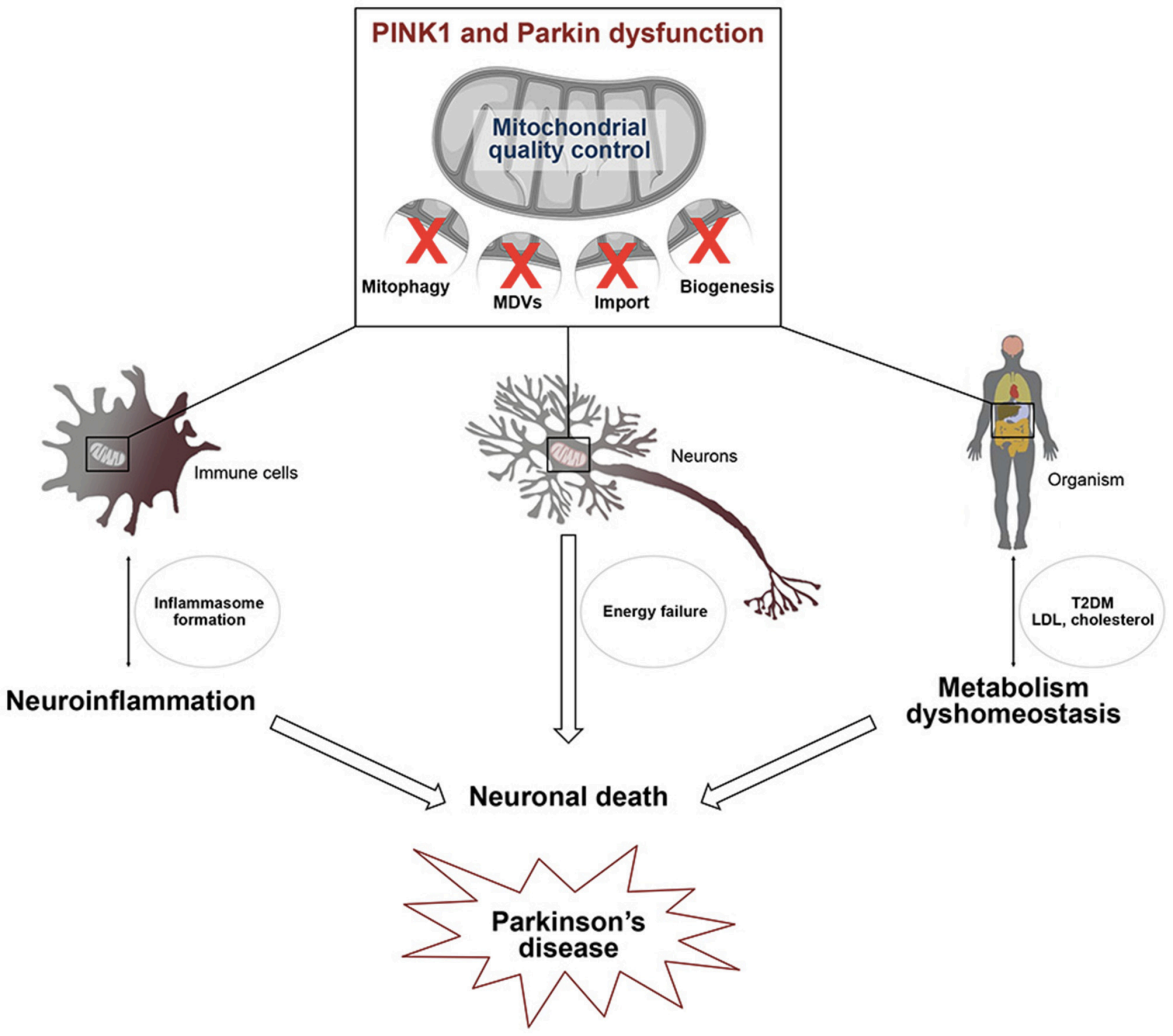
**Defects in mitochondrial quality control affect several pathways linked to Parkinson's disease**. PINK1/Parkin-dependent mitochondrial quality control, including mitophagy, the MDV pathway and mitochondrial biogenesis, is essential for cell viability. Alterations of this process in several cell types may be linked to 1/ microglial activation leading to the transcriptional induction of genes encoding components of the NLRP3 inflammasome and IL-1β 2/ energy failure in neurons, due to the presence of damaged mitochondria and 3/ type 2 diabetes (T2DM) and low density lipoprotein (LDL) cholesterol accumulation. Activation of the innate immune system, metabolism dyshomeostasis and energetic defects contribute to neuronal death which is closely linked to Parkinson's disease.

PINK1 and Parkin are not only components of a multifunctional pathway, they are also ubiquitously expressed in many cell types and organs that they may affect differently. It is thus not surprising that mutations in *PARK2* and *PARK6* have broad impacts on diverse physiological processes known or suspected to be affected in PD, including innate immunity, inflammation and metabolism (Figure 2). So far only few studies linked mechanistically neuroinflammatory or metabolic alterations associated with these mutations to dysfunction of specific mitochondrial surveillance mechanisms in definite cell types or organs. However, defects in mitophagy, the MDV pathway and PGC-1α-dependent mechanisms are emerging as central players. It is crucial that future research addresses the functional interrelationships between such defects at the intra/intercellular and organ/interorgan levels, for a more global understanding of how dysfunction of the PINK1/Parkin pathway triggers the disease. In this respect, gaining a clearer view of how PINK1/Parkin-dependent mechanisms and their impairment impact the specific biology of neurons, and more specifically those that degenerate in PD, and that of glial cells, particularly microglia and astrocytes, will provide invaluable information. Finally, future studies will have to evaluate the contribution of such mechanisms to other PD forms of genetic or sporadic origins. Following these paths will probably unravel major missing regulatory hubs and identify targets for therapeutic intervention.

## Author contributions

FML and OC wrote the manuscript. MJ and JCC have performed a critical relecture. MJ realized the graphical illustations. All authors had final approval of the submitted version.

## Funding

This work was supported by grants from Institut national de la santé et de la recherche médicale (INSERM), Fondation Institut du Cerveau et de la Moelle épinière and Agence Nationale pour la Recherche (“Investissements d'avenir,” grant ANR-10-IAIHU-06), Innovative Medicines Initiative Joint Undertaking under grant agreement n°115568, resources of which are composed of financial contribution from the European Union's Seventh Framework Programme (FP7/2007-2013) and EFPIA companies' kind contribution. Fondation de France (Grant ID Engt 2016 00066513) and the Michael J. Fox Foundation (Target Validation Awards Spring 2016 Program, Grant ID 12095).

### Conflict of interest statement

The authors declare that the research was conducted in the absence of any commercial or financial relationships that could be construed as a potential conflict of interest.
